# Effects of Single-Dose Pregabalin on Postoperative Pain in Dacryocystorhinostomy Surgery

**DOI:** 10.5812/aapm.4301

**Published:** 2012-09-13

**Authors:** Mahzad Alimian, Farnad Imani, Valiollah Hassani, Poupak Rahimzadeh, Mahshid Sharifian, Saeid Safari

**Affiliations:** 1 Department of Anesthesiology, Rasoul-Akram Medical Center, Iran University of Medical Sciences, Tehran, IR Iran

**Keywords:** Pregabalin, Pain, Postoperative, Dacryocystorhinostomy

## Abstract

**Background:**

Postoperative pain of dacryocystorhinostomy (DCA) surgery is one of the serious issues to be considered. Administrating opioids to relieve postoperative pain and facing their increasing side effects in eye surgeries, make the use of non-opioid drugs inevitable.

**Objectives:**

The present study examined the efficacy of pregabalin in alleviating the postoperative pain of DCA surgery.

**Patients and Methods:**

The present study has been carried out as a double-blind, randomized clinical trial on the patient candidates for DCR. The patients were randomly divided in to two groups of pregabalin and placebo. Patients in pregabalin group received 300 mg of pregabalin, an hour before the operation in the morning of the surgery. Pain intensity on visual analog scale (VAS) was recorded until 24 hours after the operation; also the rate of administrated opioids and nausea/vomiting frequency were recorded during the first 24-hour period after the operation and the resultsof the two groups were compared.

**Results:**

Postoperative pain intensity in the pregabalin group at the time of recovery was significantly lower than that of the placebo group (P = 0.001) until 24 hours after the surgery. In the pregabalin group 17.5% of the patients received opioids while in the placebo group the figure was 52.5% (P = 0.001). Nausea frequency was also higher in the placebo group than the pregabalin group (P = 0.003).

**Conclusions:**

A single 300 mg dose of pregabalin, an hour before DCA can effectively reduce pain intensity and also reduce opioid dose and nausea/vomiting.

## 1. Background

In spite of all the efforts of pain associations to control acute postoperative pain, the issue still looms as a serious clinical subject (1, 2). Various studies have explained undesirable impacts of unalleviated pain with maximum physiological effects on the body systems. These effects include adrenal sympathetic hyperactivity, coronary Ischemia, deep veins thrombosis, insufficient depth of breathing, atelectasis, increased heart rate, increased blood pressure, etc. (3). Although at the present time opioids are the basis for postoperative pain control, because of the known side effects of these drugs a lot of efforts aremade to reduce the need for opioid doses in relieving pain after operations by administering other drugs or practicing other methods (3-6). The ultimate purpose is to find non-opioid drugs with reasonable costs, lesser side effects and longer durability. Considering the fact that nonsteroidal anti-inflammatory drugs (NSAIDs) are administered to control the pain after eye surgeries and these drugs have side effects on kidney and digestive system, it seems that administering other drugs to alleviate patients’ pain can be helpful in dealing with the postoperative pain (7, 8). Pregabalin is an anticonvulsant drug which reduces calcium entry to the nerve terminals of central nervous system and lowers substance P, glutamate and noradrenaline levels that play roles in creating the sense of pain (9, 10). Nowadays, pregabalin is utilized in reducing neuropathic and even inflammatory pain, tissue irritation, neuralgia and fibromyalgia. We have taken into account the lesser side effects of pregabalin and its effect on acute and chronic pain, therefore it seems that this drug can be efficient in controlling mild to moderate pain of the patients after surgeries (11-15). Dacryocystorhinostomy (DCR) is among the surgeries that cause mild to moderate pain and administering opioids to relieve this level of postoperative pain leads to respiratory side effects, nausea, vomiting and urinary retention especially in old age. Administering opioids in eye surgeries increases nausea/vomiting frequency. Therefore utilizing other drugs instead of opioids can be an appropriate and practical solution in controlling the patients’ pain.

## 2. Objectives

The aim of the present study was to examine the effects of a single dose of oral pregabalin premedication (in the morning of the surgery) in alleviating DCR postoperative pain.

## 3. Patients and Methods

The present study was conducted as a double blind randomized clinical trial. Neither the subjects of study nor the persons administrating the experiment knew the critical aspects of the study. After approval of medical ethics committee and obtaining informed consent from all the patients, the candidates for DCR surgery hospitalized in the eye ward of Rasoul-Akram Hospital from 2010 to 2011 were elected through simple non-random availability sampling according to inclusion and exclusion criteria in the order of their hospitalization in the wards. The patients and the researcher were not aware of the type of intervention done in the experimental and control group .Inclusion criteria consisted of an age limit of 18 to 60 years old, being a volunteer to undergo DCR surgery, an ASA status of I or II and presenting a written consent to take part in the study. Patients with any of the following issues were excluded from the study: history of hypersensitivity to pregabalin or its derivatives, hereditary problems of galactose and glucose, lactation a medical history showing a systematic disease such as a hypertension, diabetes, collagen vascular diseases, ischemic heart diseases, kidney or liver diseases, addiction to opioids and long-term use of aspirin and other NSAIDs.

Before starting the study, a list was prepared and according to a random table totally 80 patients were placed in the two groups of A and B, each consisting of 40. Group A was the (experimental) pregabalin group and group B was the placebo group. Patients in the pregabalin (experimental) group received 300 mg of oral pregabalin an hour before entering the operation room in the morning of the surgery day (group A) while the patients in the placebo group received placebo in a similar manner (group B).

**Table:** Initial Characteristics and Pain Intensity of the Patients 24 Hours After the Operation and the Resulting Side Effects

	Pregabalin	Placebo	*P* value
Patients' age, Mean ± SD	41.1 ± 14.1	45.4 ± 15.7	0.212
Frequency of men, frequancy (%)	28 (70)	21 (52.5)	0.108
Pain intensity at the time of recovery, Mean ± SD	3.2 ± 1.5	5.1 ± 1.5	< 0.001
Pain intensity 30 min after the operation, Mean ± SD	3.1 ± 1.6	5 ± 1.4	< 0.001
Pain intensity 2 hours after the operation, Mean ± SD	2.7 ± 1.4	5.4 ± 1.6	< 0.001
Pain intensity 4 hours after the operation, Mean ± SD	2.5 ± 1.5	5.1 ± 1.7	< 0.001
Pain intensity 12 hours after the operation, Mean ± SD	1.02 ± 1	3.2 ± 1.6	< 0.001
Pain intensity 24 hours after the operation, Mean ± SD	0.6 ± 0.8	1.6 ± 1.5	< 0.001
Nausea, frequency (%)	5 (12.5)	17 (47)	0.003
Vomiting, frequency (%)	1 (2.5)	5 (12.5)	0.09

The anesthesiologist in the operation room had no information about the premedication of the patients. After establishing intravenous line and standard monitoring, 5cc/kg of ringer solution was infused. Fentanyl 3 µ/kg and midazolam 1 mg was injected as premedication and for induction of anesthesia thiopental 4 mg/kg and of atracurium 0.5 mg/kg were injected and for maintenance of anesthesia propofol 100 µg/kg/min and atracurium 10 mg (every 30 minutes) and fentanyl 1 µg/kg (every 45 minutes) were infused. In the last 30 minutes of the operation injecting of opioids was prohibited. On arrival to the recovery room and 30 minutes, 1 hour, 4 hour, 12 hours and 24 hours after the operation pain intensity was measured based on visual analog scale (VAS). At the end of the 24-hour period opioids administered to relieve patients` pain were recorded and during this period nausea/vomiting occurrences were checked in the both groups. For the patients whose pain intensity exceeded three on VAS measurement, 25 mg pethedine was administered intramuscularly and documented. The data gathered from the study were analyzed using statistical analysis software (SPSS 16). Quantitative data were presented as the mean and standard deviation and qualitative data were presented as frequency and percentage frequency. In order to compare the quantitative data between the two groups the student t-test statistical method was used according to normal distribution. To compare qualitative data between the two groups k-2 statistical method was used. The variances were analyzed to determine the precise effect and omit the effect of frequent measurements, at 0.5 level.

## 4. Results

40 patients in the pregabalin (experimental) group and 40 patients in the placebo group were under study. The age and sex distribution of the patients are presented in Table. According to the Table the two groups are similar in age distribution and there is no statistically significant difference between the two groups (P = 0.212). Most of the patients under study were men (49 people, 61.2%). The sex distribution is also similar in both groups and shows no statistically significant difference (P = 0.108). The pain intensity mean of pregabalin (experimental) group in recovery was 3.2 ± 1.5 which was statistically lower than that of placebo group (5.1 ± 1.5) (P = 0.001, Figure). In the pregabalin group, seven patients (17.5%) needed opioids to relieve postoperative pain while this figure was 21 people (52.5%) in the placebo group (P = 0.001). The probability of taking drugs in the placebo group was 5.2 times more than that of pregabalin group (confidence interval) 95% equal to (1.87 to 14.5). All the patients had single injection of opioids. Nausea frequency in the pregabalin group was 12.5% (five patients) and 42.5% (17 patients) in the placebo group which is statistically significant (P = 0.003). Vomiting frequency of the pregabalin group was 2.5% (one patient) and in the placebo group the figure was 12.5% (five patients) (P = 0.09). None of the patients complained about other side effects such as: dizziness, sedation, headache or visionary disorder in this study.

**Figure:**
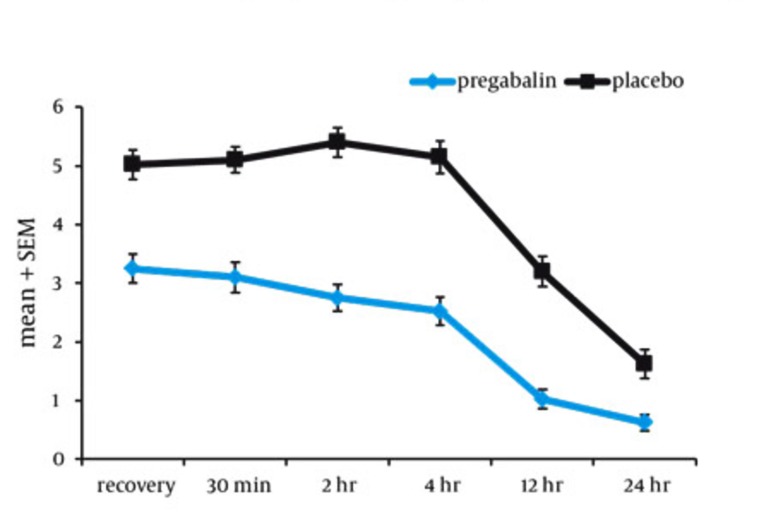
Pain Intensity Variations (Based on VAS) in the Patients Under Study During 24 Hours After the Operations

## 5. Discussion

Postoperative pain is naturally sensed by nociceptive receptors. However, the trauma of the operation can cause hyperalgesia which prolongs the postoperative pain. Unlike traditional analgesics which are nociceptive, gabapentoids like gabapentin and pregabalin decrease the stimulation of posterior horn neurons caused by tissue damage compared to the period before afferent entry from tissue damage spot. On this basis gabapentoids (administered before the operation) are recommended to relieve acute surgery pain (15). In recent years, pregabalin has been used as an adjuvant drug in dealing with postoperative pain (10, 13). Frequent studies have been carried out to examine the efficacy and side effects of pregabalin in relieving pain but the results have been contradictory and because of differences in doses, administration, and other innate differences in each of the operations it is difficult to generalize the results. Hill and his colleagues examined the effect of administrating two 50 and 300 mg doses of pregabalin after the operation on postoperative pain of dental surgery and indicated that administrating 300 mg of pregabalin can significantly reduce the postoperative pain although the side effects in this group exceeded the side effects of placebo and 50 mg pregabalin group (16). Paech and his colleagues tried to study the effect of administrating pregabalin 100 mg (an hour before the gynecological operations and general anesthesia) on alleviating postoperative pain. The results of this study indicated that there is no significant difference in the pain intensity between the two groups despite the fact that intravenous fentanyl had been injected for analgesia after the operation in both groups (17). On the other hand side effects like mild headache, visionary disorders and walking problems occurred more in the pregabalin group than the placebo group. Agrawal and his colleagues administered pregabalin 150 mg an hour before the operation to alleviate the postoperative pain of laparoscopic cholecystectomy under general anesthesia. In this clinical trial the pain intensity of the pregabalin group was significantly lower than the placebo group though fentanyl had been injected in the veins of both groups (18). Comparing these studies with our survey indicates that in certain types of surgeries which tissue damage is not extensive and results in less mediator release, and the dosage of pregabalin are two important factors in reducing pain intensity, prescribing 300 mg of oral pregabalin in the morning of surgery in this study effectively and notably alleviated the postoperative pain of surgeries with mild to moderate postoperative pain like DCR. However there is disagreement regarding the efficacy of pregabalin in major surgeries such as abdominal surgeries. Jokela and his colleagues investigated the various doses of pregabalin (150, 300 and 600 mg) in laparoscopic cholecystectomy and hysterectomy in two different studies (19, 20). The work of this group indicated that administering 300 mg Pregabalin before the surgery and repeating it 12 hours after the operation had the most remarkable effect on the postoperative pain but the side effects ( such as dizziness) appeared more in this dose. Nevertheless, Chang and his colleagues also used this dose in the form of two separate doses, one 150 mg dose before the operation and one 150 mg dose 12 hours after the operation. They found no significant difference in the pain intensity of cholecystectomy patients between the two groups after the operation (21). Mathiesen and his colleagues also carried out two different studies on the patients undergone painful surgeries such as hip arthroplasty and abdominal hysterectomy (22, 23). One systematic review carried out by Zhang and his colleagues collected all reliable clinical trials to investigate the efficacy of pregabalin in alleviating postoperative pain (24). This meta-analysis showed that administering Pregabalin in doses less than 300 mg before the operation cannot significantly alleviate the pain in the first 24-hour period after the surgery while increasing the dose can significantly decrease the pain intensity and amplify the side effects resulting from pregabalin. In our study prescribing 300 mg of oral pregabalin in the morning of surgery caused no side effects and effectively reduced the postoperative pain which indicates single oral dose of pregabalin in 300 mg per dose is more effective than doses under 300 mg or in divided doses compared to other studies. However, type of surgery is an important factor and in minor to moderate surgeries pregabalin has its maximal effect without any side effects.

The results of this study indicated that a single dose of pregabalin 300 mg an hour before DCR operation can significantly reduce the postoperative pain and decrease opioid doses compared to the placebo group. Also nausea/vomiting frequency was lower in the pregabalin group without any particular side effects. Reduced nausea/vomiting frequency after postoperative pregabalin administration accord with the results of other studies but unlike other reports about pregabalin side effects such as dizziness, sedation, headache, and visionary disorder, none of the patients in this study complained about these side effects. One of the limitations of the study was that the sedation score was not evaluated. We recommend that in future studies this score be evaluated and comparison of pregabalin alone with combined regime such as pregabalin and NSAIDs be studied.

## References

[1] Kehlet H, Dahl JB (2003). Anaesthesia, surgery, and challenges in postoperative recovery.. Lancet..

[2] Moradi M, Esmaeili S, Shoar S, Safari S (2012). Use of Oxycodone in Pain Management.. Anesth Pain..

[3] Miller RD (2005). Anesthesia..

[4] Dell R (1996). A review of patient-controlled sedation.. Eur J Anaesthesiol..

[5] Imani F, Safari S (2011). Pain Relief is an Essential Human Right”, We Should be Concerned about It.. Anesth Pain..

[6] Shoar S, Esmaeili S, Safari S (2012). Pain Management After Surgery: A Brief Review.. Anesth Pain..

[7] Kehlet H, Dahl JB (1992). Are perioperative nonsteroidal anti-inflammatory drugs ulcerogenic in the short term?. Drugs..

[8] Moote C (1992). Efficacy of nonsteroidal anti-inflammatory drugs in the management of postoperative pain.. Drugs..

[9] Ben-Menachem E (2004). Pregabalin pharmacology and its relevance to clinical practice.. Epilepsia..

[10] Alimian M, Imani F, Faiz SHR, Pournajafian A, Navadegi SF, Safari S (2012). Effect of Oral Pregabalin Premedication on Post-Operative Pain in Laparoscopic Gastric Bypass Surgery.. Anesth Pain..

[11] Chizh BA, Gohring M, Troster A, Quartey GK, Schmelz M, Koppert W (2007). Effects of oral pregabalin and aprepitant on pain and central sensitization in the electrical hyperalgesia model in human volunteers.. Br J Anaesth..

[12] Frampton JE, Foster RH (2005). Pregabalin: in the treatment of postherpetic neuralgia.. Drugs..

[13] Gilron I (2007). Gabapentin and pregabalin for chronic neuropathic and early postsurgical pain: current evidence and future directions.. Curr Opin Anaesthesiol..

[14] Guay DR (2005). Pregabalin in neuropathic pain: a more “pharmaceutically elegant” gabapentin?. Am J Geriatr Pharmacother..

[15] Shneker BF, McAuley JW (2005). Pregabalin: a new neuromodulator with broad therapeutic indications.. Ann Pharmacother..

[16] Hill CM, Balkenohl M, Thomas DW, Walker R, Mathe H, Murray G (2001). Pregabalin in patients with postoperative dental pain.. Eur J Pain..

[17] Paech MJ, Goy R, Chua S, Scott K, Christmas T, Doherty DA (2007). A randomized, placebo-controlled trial of preoperative oral pregabalin for postoperative pain relief after minor gynecological surgery.. Anesth Analg..

[18] Agarwal A, Gautam S, Gupta D, Agarwal S, Singh PK, Singh U (2008). Evaluation of a single preoperative dose of pregabalin for attenuation of postoperative pain after laparoscopic cholecystectomy.. Br J Anaesth..

[19] Jokela R, Ahonen J, Tallgren M, Haanpaa M, Korttila K (2008). Premedication with pregabalin 75 or 150 mg with ibuprofen to control pain after day-case gynaecological laparoscopic surgery.. Br J Anaesth..

[20] Jokela R, Ahonen J, Tallgren M, Haanpaa M, Korttila K (2008). A randomized controlled trial of perioperative administration of pregabalin for pain after laparoscopic hysterectomy.. Pain..

[21] Chang SH, Lee HW, Kim HK, Kim SH, Kim DK (2009). An evaluation of perioperative pregabalin for prevention and attenuation of postoperative shoulder pain after laparoscopic cholecystectomy.. Anesth Analg..

[22] Mathiesen O, Jacobsen LS, Holm HE, Randall S, Adamiec-Malmstroem L, Graungaard BK (2008). Pregabalin and dexamethasone for postoperative pain control: a randomized controlled study in hip arthroplasty.. Br J Anaesth..

[23] Mathiesen O, Rasmussen ML, Dierking G, Lech K, Hilsted KL, Fomsgaard JS (2009). Pregabalin and dexamethasone in combination with paracetamol for postoperative pain control after abdominal hysterectomy. A randomized clinical trial.. Acta Anaesthesiol Scand..

[24] Zhang J, Ho KY, Wang Y (2011). Efficacy of pregabalin in acute postoperative pain: a meta-analysis.. Br J Anaesth..

